# Silencing of hepatic fate-conversion factors induce tumorigenesis in reprogrammed hepatic progenitor-like cells

**DOI:** 10.1186/s13287-016-0349-5

**Published:** 2016-07-27

**Authors:** Felipe Serrano, Maria García-Bravo, Marina Blazquez, Josema Torres, Jose V. Castell, Jose C. Segovia, Roque Bort

**Affiliations:** 1 Unidad de Hepatología Experimental, Instituto de Investigación Sanitaria La Fe, CIBERehd, Avda. Fernando Abril Martorell 106, 46026 Valencia, Spain; 2 Differentiation and Cytometry Unit, Division of Hematopoietic Innovative Therapies, Centro de Investigaciones Energéticas, Medioambientales y Tecnológicas (CIEMAT) and Centro de Investigación Biomédica en Red de Enfermedades Raras (CIBER-ER), Instituto de Investigación Sanitaria Fundación Jiménez Díaz (IIS-FJD, UAM), Avda. Complutense 40, 28040 Madrid, Spain; 3 Departamento de Biología Celular, Universidad de Valencia, Avda. Doctor Moliner 50, 46100 Burjassot, Spain; 4 Present address: Anne McLaren Laboratory for Regenerative Medicine and Wellcome Trust-Medical Research Council, Cambridge Stem Cell Institute, University of Cambridge, West Forvie Site, Robinson Way, Cambridge, CB2 0SZ UK

**Keywords:** Direct reprogramming, Hepatocyte, Progenitor, Tumorigenesis, Xenograft

## Abstract

**Background:**

Several studies have reported the direct conversion of mouse fibroblasts to hepatocyte-like cells with different degrees of maturation by expression of hepatic fate-conversion factors.

**Methods:**

We have used a combination of lentiviral vectors expressing hepatic fate-conversion factors with *Oct4*, *Sox2*, *Klf4*, and *Myc* to convert mouse embryonic fibroblasts into hepatic cells.

**Results:**

We have generated hepatic cells with progenitor-like features (iHepL cells). iHepL cells displayed basic hepatocyte functions but failed to perform functions characteristic of mature hepatocytes such as significant Cyp450 or urea cycle activities. iHepL cells expressed multiple hepatic-specific transcription factors and functional genes characteristic of immature hepatocytes and cholangiocytes, as well as high levels of *Foxl1*, *Cd24a*, and *Lgr5*, specific markers of hepatic progenitor cells. When transplanted into partial hepatectomized and hepatic irradiated mice, they differentiated into hepatocytes and cholangiocytes. However, iHepL cells formed malignant non-teratoma cell aggregations in one out of five engrafted livers and five out of five xenografts assays. All the cells in these tumors had silenced key hepatic fate-conversion factors, and lost hepatic features.

**Conclusions:**

This study highlights the dangers of using pluripotency factors in reprogramming strategies when fate-conversion factors are silenced in vivo, and urges us to perform extensive tumorigenic tests in reprogrammed cells.

**Electronic supplementary material:**

The online version of this article (doi:10.1186/s13287-016-0349-5) contains supplementary material, which is available to authorized users.

## Background

Overexpression of lineage-specific transcription factors directly converts fibroblasts into other cell types such as neurons [1], cardiomyocytes [2], blood progenitors [3], or hepatocytes [4–7]. Hepatocyte-like cells obtained in different reports, either by direct conversion of fibroblasts or indirectly by differentiation of induced pluripotent stem cells (iPSC), display a wide range of drug-metabolizing genes as well as genes involved in hepatocyte metabolic functions, reflecting a wide variety of maturation [8].

On the other hand, terminally differentiated cells cannot proliferate in vitro, limiting their utility in biotechnology and regenerative medicine [1, 4, 5]. Thus, we have focused on reprogramming fibroblasts into expandable progenitor cells capable of differentiating into different cell types within the same lineage. We reasoned that concomitant expression of *Oct4*, *Sox2*, *Klf4*, and *Myc* (OSKM) together with cell fate-converting transcription factors could maintain cells in a stem-like fashion allowing their proliferation and differentiation when exposed to the appropriate extracellular cues. In fact, induced hepatic stem cells (iHepSC) generated from mouse fibroblasts are phenotypically closer to fetal hepatocytes than mature hepatocytes, and they only achieve full maturation after transplantation into FRG mice [9]. Having stated the advantages of reprogramming into progenitor-like cells, it should also be highlighted that inclusion of *Myc* in reprogramming cocktails boost reprogramming, but increases the possibility of obtaining cells prone to tumorigenicity.

In our study, we have obtained bipotential hepatic progenitor-like (iHepL) cells by expression of reprogramming factors together with hepatic fate-conversion factors. We selected *Hnf4a*, *Hnf1a*, and *Foxa2* since they act coordinately to control multiple aspects of hepatocyte differentiation, liver development, and function [10]. *Hhex* is expressed in the early hepatic endoderm during liver development in mice [11]. Gata factors are crucial for competency of the definitive endoderm [12], and *Hnf6a* absence results in premature differentiation of biliary cells [13]. Our iHepL cells do not express pluripotency markers, but they express high levels of two hepatic progenitor-specific genes, *Foxl1* and *Cd24a* [14, 15], as well as markers of ductal cells. When transplanted in vivo, those progenitor cells are able to differentiate into hepatocytes and cholangiocytes. However, the cells form tumors in xenograft assays when hepatic fate-conversion factors are spontaneously silenced.

## Methods

### Cell media and imaging

Mouse embryonic fibroblasts (MEF) were prepared from 13.5-day post-coitum embryos. MEF were grown in DMEMc (Dulbecco’s modified Eagle’s medium (DMEM) supplemented with 10 % fetal bovine serum (FBS) and 2 mM Glutamax). In the reprogramming experiments two different media were used: hepatocyte conditioned medium (HCM) I and HCM II. HCM I is composed of IMDM:F12 (1:3), supplemented with 10 % FBS, 2 mM l-glutamine, penicillin/streptomycin, 10 ng/ml epidermal growth factor (EGF), 100 ng/ml fibroblast growth factor (FGF)2, 50 ng/ml vascular endothelial growth factor (VEGF), and 100 ng/ml transforming growth factor (TGF)β. HCM II is composed of IMDM:F12 (1:3), supplemented with 10 % FBS, 2 mM l-glutamine, penicillin/streptomycin, 10 ng/ml hepatocyte growth factor (HGF), and 10 ng/ml Oncostatin M. All media was purchased from Invitrogen (www.thermofisher.com). Growth factors were purchased from R&D Systems (www.rndsystems.com). iHepL cells exhibited enhanced attachment to the culture dishes and needed trypsinization for 30 min at 37 °C for passaging.

All cells were maintained at 37 °C with 5 % CO_2_ and were regularly examined with an Olympus CKX41 microscope. Images were taken on an Olympus FV1000 confocal mounted on an IX81 inverted microscope.

### Plasmids and retrovirus generation

The retroviral constructs pMIGR1-Hhex, pMIGR1-Hnf1a, and pMIGR1-Hnf6a were generated by polymerase chain reaction (PCR) amplification of the cDNAs (see Additional file 1: Table S1 for oligo sequence) followed by subcloning into the XhoI-EcoRI restriction sites of pMIGR1 [16]. All constructs were verified by sequencing. pBabe-Foxa2, pBabe-Hnf4a, and pBabe-Gata4 are derivatives of the pBabe-puro retroviral vector [17] donated by Dr. Ken Zaret (University of Pennsylvania, Philadelphia, PA, USA). The plasmids encoding the reprogramming factors pMXs-Oct4, pMXs-Sox2, pMXs-Klf4, and pMXs-cMyc were purchased from Addgene (Cambridge, MA, USA; www.addgene.com) [18]. A summary of the retroviral plasmids is shown in Additional file 1 (Table S2). Ecotropic retroviruses were generated in 293 T cells as described elsewhere [19]. MEF were infected with equal volumes of each retrovirus.

### Primary hepatocyte isolation and culture

Mice hepatocytes were isolated using a two-step perfusion technique as previsouly described [20]. Briefly, the liver was pre-perfused through the portal vein with calcium-free buffer (118 mM NaCl, 4.7 mM KCl, 1.2 mM H_2_KPO_4_, 1.2 mM Mg_2_SO_4_, 25 mM HNaCO_3_, 10 mM glucose, 0.5 mM EGTA, pH 7.4) and then perfused with the same buffer containing 2.5 mM CaCl_2_ and 125 U/ml collagenase IV. Once the enzymatic digestion was completed, the liver was transferred to a petri dish and the cells were gently dispersed with a blunt tool. Cells were collected by low-speed centrifugation. Viability of isolated hepatocytes was around 90 % as determined by Trypan blue.

### iHepL induction

Approximately 10^6^ early passage (passage 2 or 3) MEF were seeded on a 10-cm dish containing DMEMc. One day later, cells were infected with indicated viruses supplemented with 4 μg/ml polybrene for 24 h. Seventy two hours later, media were changed to HCM I and the culture continued for an additional 18 days. Culture medium was refreshed every day. Cells were frozen at this stage at 1.5 × 10^6^ cells per vial to generate cell stocks for future use. The final differentiation step was achieved by thawing one vial into a 10-cm dish containing HCM II media with further culturing for 6 days. Cells should be split if confluent by using TrypLE™ Select. If needed, cells can be maintained for longer periods by regular passaging. Phenotypic characterization of iHepL cells was performed at confluency. All the data shown in the paper come from the average of two biological replicates from each of the three infections.

To obtain isogenic cell clones, cells were seeded at low density and colonies were transferred to 96-well plates and expanded. Data depicted in the figures in Additional file 1 were obtained from two biological replicates from clone 2 (isolated from infection #1), clone 6 (isolated from infection #2), and clone 15 (isolated from infection #3).

### Reverse transcription and quantitative PCR

Total RNA was extracted using the RNeasy mini kit (www.qiagen.com) and reverse-transcribed using Moloney Murine Leukemia Virus reverse transcriptase (www.thermofisher.com) according to the manufacturer’s protocol. Genomic DNA was extracted from paraffin-embedded tumors and tissues using the Qiamp DNA FFPE Tissue Kit (www.qiagen.com). PCR amplification was performed using the expand high fidelity PCR system (Roche, Basel, Switzerland; www.lifescience.roche.com) following the manufacturer’s instructions on a Light Cycler 480 II Real-Time PCR System using the Light Cycler 480 SYBR Green I Master. The specificity of the amplified PCR products was confirmed by analysis of the melting curve and agarose gel electrophoresis. Primers used for the quantitative PCR (qPCR) are shown in Additional file 1: Tables S3 and S4. Primers designed to measure endogenously expressed transcription factors were validated by running a PCR assay using the corresponding retroviral shuttle plasmids. The relative expression of each mRNA was normalized against mouse beta Actin (Actb). The relative gene copy number in genomic DNA was estimated as described elsewhere [21] using a fragment amplified from intron 1 of Ccnd1for normalization. Primers used to amplify exogenous genes were validated using the corresponding retroviral plasmids.

MicroRNA was extracted with an adapted protocol from Qiagen using the miRNeasy kit which consisted of precipitating with 1.5 volumes of 100 % ethanol instead of 1 volume of 70 % ethanol. miR122-specific reverse transcription stem loop was performed using the primer 5′-GTCGTATCCAGTGCAGGGTCCGAGGTATTCGCACTGGATACGACCAAACA-3′ as described in [22]. qPCR was run using miR-122 specific primers 5′-TTGGAGCTCCCTTTTTGCTA-3′ and 5′-CACCATGCCTGGCTAATTTT-3′.

### Cell and tissue immunofluorescence

Cells were fixed with 4 % paraformaldehyde at room temperature, and then washed three times with phosphate-buffered saline (PBS). Cells were blocked with blocking buffer (5 % normal donkey serum, 0.3 % Triton X-100 in PBS) for 60 min at room temperature and then incubated with primary antibodies at 4 °C overnight in 0.1× blocking buffer. The next day cells were washed three times with PBS, and then incubated with Alexa Fluor conjugated secondary antibodies at 1/500 dilution (Life Technologies) for 60 min at room temperature in the dark. Nuclei were stained with DAPI (200 ng/ml for 15 min). The high content screening imaging station Scan® from Olympus was used to analyze the number and intensity of cell staining. Total cell number was calculated from the number of DAPI-positive nuclei (software used).

Sections of the paraffin-embedded tissues were dewaxed and rehydrated through ethanol:water series and unmasked by boiling in sodium citrate buffer (10 mM, pH 6.0, for 10 min). Immunostaining was performed as described above, but the blocking buffer contained 10 % normal donkey serum in PBT (0.1 % Tween 20 in PBS). Slides were mounted with fluorescence mounting media (Dako). Antibodies used for immunofluorescence staining are described in Additional file 1 (Table S5).

### Flow cytometry

Adherent cells were washed with PBS and detached by treatment with trypsin (Life Technologies). Cells fixed with 4 % paraformaldehyde for 10 min at 4 °C were permeabilized with 90 % methanol for 30 min at 4 °C. After blocking with 0.5 % ovalbumin, cells were incubated with the primary antibody or normal serum (control) for 60 min at room temperature. Cells were then incubated with secondary antibody for 30 min in the dark. Cells were analyzed by FACSCanto Flow Cytometer (Becton Dickinson). Nuclei were stained with DAPI (200 ng/ml in PBS) for 15 min in the dark. Primary antibodies used for flow cytometry are described in Additional file 1 (Table S5). To determine the percentage of live green fluorescent protein (GFP)-positive cells, trypsinized cells were resuspended in complete DMEM containing 50 μg/ml 7-amino actinomycin D (7-AAD) and incubated for 30 min in the dark. Data were analyzed using FACSDiva software (Becton Dickinson). Doublets were eliminated using a pulse geometry gate (FSC-H x FSC-A).

### Indocyanine green uptake, PAS staining, glycogen depletion, Cyp450 metabolism, and Ugt activity

Confluent cells were incubated with fresh HCM II medium supplemented with 1 mg/ml indocyanine green at 37 °C for 30 min. Once pictures were taken, cells were washed with PBS and cultured in media without indocyanine green. Cells were stained by periodic acid-Schiff (PAS; Sigma) following the manufacturer’s instructions. To induce glycogen depletion by starvation, iHepL cells cultured in six-well plates were incubated with Krebs-Henseleit solution without glucose. Cells were fixed at 0, 1.5, 3, and 6 hours and stained with PAS to estimate intracellular glycogen content. To induce glycogen depletion with glucagon, cells were incubated in HCM II media containing 300 ng/ml glucagon.

Cyp450 activities were measured essentially as described previously [23]. Briefly, confluent iHepL cells cultured in six-well plates were incubated in media containing eight substrates for 4 h at 37 °C. Metabolites were measured in the supernatant by liquid chromatography mass spectrometry (LC-MS/MS). Total cell protein was used to normalize the data. Isolated primary hepatocytes and wild-type MEF were used as a positive and negative control, respectively. Formation of estradiol-3-glucuronide and estradiol-17-glucuronide was measured in cells incubated with β-estradiol 100 μM for 2 h as described previously [24].

### Cell transplantation into partial hepatectomized and hepatic irradiated mice

The Animal Care and Ethics Committee from CIEMAT specifically approved this study (approval number HEM4-12). The mouse strain used was NSG (NOD.Cg-Prkdc^scid^ Il2rg^tm1Wjl^/SzJ). Anesthesia was induced by intraperitoneal injection of a mixture of ketamine (125 mg/kg) and medetomidine (10 mg/kg). To provide a proliferative stimulus to hepatic cells, a 45 % partial hepatectomy (PH) was conducted, removing the right and left lateral lobules through a transversal midline incision in the abdomen of NSG mice. Immediately after PH, the animals were placed in a supine position inside a lead box where a hole was made to allow the localized irradiation of the median hepatic lobule. Two 3 × 5-cm lead shields, each 2 mm thick, were fitted under the liver to protect the stomach and intestines. The liver was irradiated using Philips MG324 X-ray equipment (Philips, Hamburg, Germany) to deliver a dose of 41.05 Gy, 260 kV, 12.3 mA, at a dose rate of 4.105 Gy/min. The abdomen was then closed in two layers. Anesthesia was reverted by intraperitoneal injection of atipamezol (50 mg/kg). Cell transplantation was performed 4 days after PH and hepatic irradiation. Mice were anesthetized and the spleen was exposed by a left flank incision. Homeostasis was achieved by ligation of the splenic tip using a 2-0 silk suture. To validate our model, 10^6^ hepatocytes freshly isolated by collagenase perfusion of the liver from ubiquitously expressing mRFP mice were injected (data not shown). In subsequent experiments, 10^6^ iHepL cells were injected. Three weeks after transplantation, mice were sacrificed and pieces of the liver were fixed in formalin and histologically evaluated after paraffin-embedding.

### Subcutaneous xenograft animal model

Male, 6- to 8-week-old NSG (NOD.Cg-Prkdc^scid^ Il2rg^tm1Wjl^/SzJ) were kept at least 1 week before experimental manipulation. Cultured iHepL cells (1.5 × 10^6^/injection) in 150 μl DPBS were implanted subcutaneously in the right flank region of mice (*n* = 5). Tumor progression was followed for up to 6 weeks. Upon sacrifice, primary tumors were removed, formalin-fixed, and histologically evaluated.

### Statistical analysis

All in vitro experiments were conducted a minimum of three times. Data are expressed as the mean ± standard deviation. Statistical significance was calculated by unpaired Student’s *t* test.

## Results

### Co-expression of hepatic transcription factors with OSK/M allows transdifferentiation of MEF to hepatic-like cells without pluripotent commitment

Considering that reprogramming is a stochastic process, we reasoned that initial cell reprogramming by expression of OSK/M could be diverged towards the hepatic cell fate by co-expressing key determinants of hepatic cell fate [25]. MEF were infected with retroviral vectors expressing *Gata4*, *Foxa2*, *Hnf4a*, *Hhex*, *Hnf1a*, and *Hnf6a*, with or without OSK/M, and cultured with DMEMc for 2 days. Then, cells were split into two gelatine-coated 10-cm plates; one plate was maintained in DMEMc and the other in HCM I containing essential growth factors for maintenance of hepatic lineage. Leukemia inhibitory factor (LIF) was not included in the reprogramming media to hamper commitment to the pluripotent fate.

**Fig. 1:**
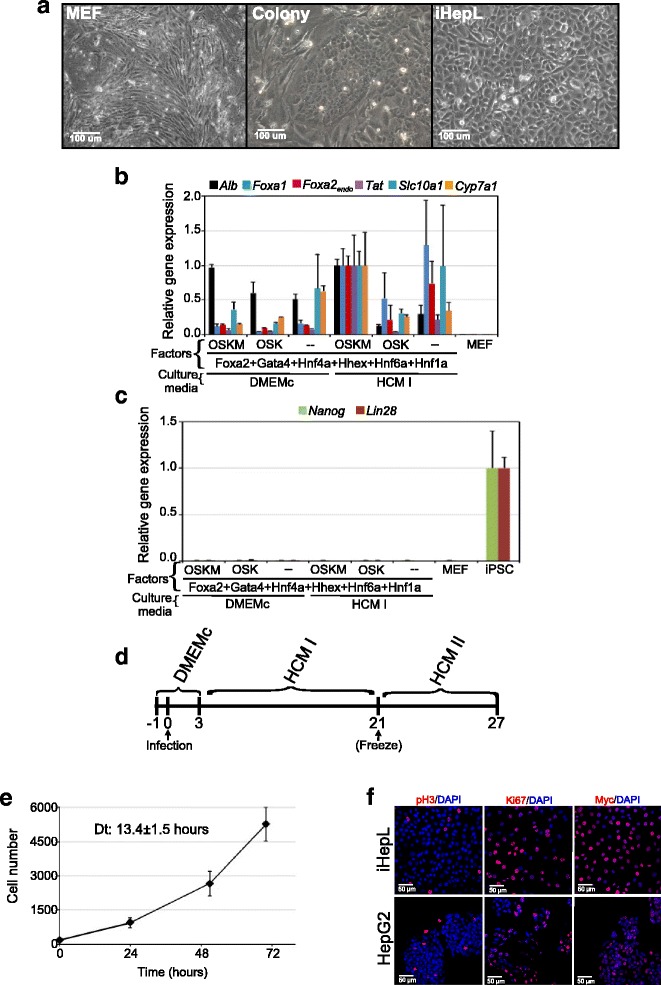
Conversion of MEF into hepatic-like cells. **a** Phase contrast photographs of mock-infected mouse embryonic fibroblasts (*MEF*), an epithelial colony (7 days after infection; *Colony*), and early-stage hepatic progenitor-like (*iHepL*) cells. **b** Expression levels of *Alb*, *Foxa1*, *Foxa2*_*endo*_, *Tat*, *Slc10a1* (Ntcp), and *Cyp7a1* mRNA in pooled MEF at 14 days post-infection grown under the indicated conditions. Mock-infected MEF grown in DMEMc were used as negative control. OSKM: *Oct4*, *Sox2*, *Klf4*, *Myc*; OSK: *Oct4*, *Sox2*, *Klf4*. Data are represented as mean ± SD (*n* = 3). **c** Expression levels of *Nanog* and *Lin28* mRNA as described in (b). MEF infected with OSKM (iPSC) were used as a positive control. **d** Timeline showing the improved experimental procedure for direct induction of hepatic-like cells. **e** Growth curve of iHepL cells at day 21. Exponential growth accurately described the data (R^2^ above 0.99). The population-averaged doubling time (*Dt*) was calculated from these fits. Data are represented as mean ± SD (*n* = 3). **f** Representative fluorescence images of HepG2 and iHepL cells immunostained for proliferative markers phospho-Histone H3 (pH3), Ki67, and Myc. Nuclei were stained with DAPI

When looking at the reprogramming plates at day 7, we identify groups of epithelial cells with flattened, enlarged, and polygonal shapes between fibroblasts (Fig. 1a and Additional file 1: Figure S1A). The morphology of iHepL cells resembled that of hepatic endoderm, not mature polygonal hepatocytes. At later stages, cells with fibroblastic morphology died out while the cultures became homogeneously formed by epithelial cells. We extracted RNA at day 14 to check if any plate had initiated the hepatic differentiation program by measuring the expression of early hepatic markers *Alb*, *Foxa1*, *Foxa2*_endo_, *Tat*, *Slc10a1* (Ntcp), and *Cyp7a1* (Fig. 1b). The combination of hepatic factors and OSKM supplemented with the incubation of the transduced cells in HCM I showed the best induced hepatic-gene expression (Additional file 1: Figure S1B). Concomitantly, we did not detect expression of *Nanog* and *Lin28*, confirming that infected cells did not commit to the pluripotent fate (Fig. 1c). It is noteworthy that retroviral combinations that did not include OSK/M became quiescent and did not allow us to obtain frozen cellular stocks. Thus, the presence of reprogramming factors in the cocktail not only seems to improve the expression of early hepatic markers, but also provides the proliferative stimuli necessary for both reprogramming and cell growth [26]. We selected the retroviral cocktail containing OSKM with *Gata4*, *Foxa2*, *Hnf4a*, *Hhex*, *Hnf1a*, and *Hnf6a* for future improvement of our differentiation protocol.

### iHepL cells repress fibroblast markers and activate the hepatic transcriptional program

Having successfully initiated the earliest hepatocyte reprogramming of MEF, we added an additional step in order to improve our differentiation protocol (Fig. 1d). Briefly, infected MEF were cultured in DMEMc for 3 days and then cells were switched to HCM I and maintained for 18 days. At this stage, cells could be maintained in culture at low density for several passages in HCM I media without any further differentiation (expansion phase), frozen in liquid nitrogen, or continue to the final differentiation process. The efficiency of conversion to early iHepL was approximately 0.06 %. To induce terminal differentiation, cells (frozen or in culture) were cultured at high confluence in HMC II media for 6 additional days. Early iHepL cells (after 21 days) were highly proliferative in culture (Fig. 1e), probably due to the expression of *Myc* (Fig. 1f).

**Fig. 2:**
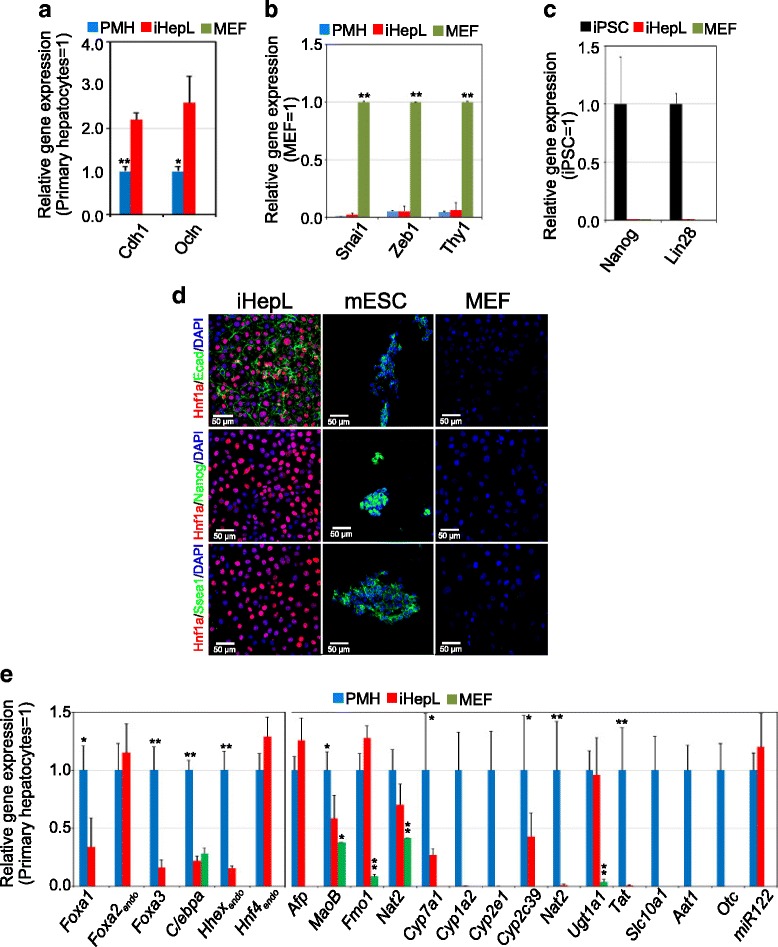
iHepL cells upregulate hepatic mRNA. Total RNA was extracted from hepatic progenitor-like (*iHepL*) cells, mouse embryonic fibroblasts (*MEF*), primary cultured mouse hepatocytes (*PMH*) and induced pluripotent stem cells (*iPSC*). Expression levels of epithelial markers (**a**), mesenchymal markers (**b**), and pluripotency markers (**c**) were assessed by qRT-PCR and plotted as relative gene expression. Data are represented as mean ± SD from iHepL cells (*n* = 6; three infections and two biological replicates each), MEF (*n* = 3), PMH (*n* = 4), and iPSC (*n* = 3). Student’s *t* test was performed between iHepL and the other groups; *p < 0.05; **p < 0.01. **d** Representative fluorescence images of iHepL, mouse embryonic stem cells (*mESC*), and MEF immunostained for E-cadherin and the pluripotency markers Nanog and Ssea-1. Nuclei were stained with DAPI. **e** mRNA levels of multiple hepatic markers were measured by qRT-PCR in total RNA extracted from MEF and iHepL cells. Data are represented as mean ± SD from iHepL cells (*n* = 6; three infections and two biological replicates each), MEF (*n* = 3), and PMH (*n* = 4). Student’s *t* test was performed between iHepL and the other groups; *p < 0.05; **p < 0.01

iHepL cells displayed a typical epithelial morphology, showing a significant increase in the epithelial markers *Cdh1* and *Ocln* (Fig. 2a and d) and downregulation of the mesenchymal markers *Snail1*, *Zeb1*, and *Thy1*(Fig. 2b).

Our protocol is not favorable for the establishment or maintenance of pluripotency since we do not include LIF in our reprogramming media. However, to exclude the possibility that we carried over cells from colonies in an intermediate reprogramming state, we measured the expression levels of critical genes in the pluripotent transcriptional network. We could not detect expression of *Nanog*, *Lin28*, endogenous *Oct4*, or endogenous *Sox2* by qRT-PCR (Fig. 2c and Additional file 1: Figure S2A). Moreover, exogenous *Oct4* and *Sox2* could not be detected. Absence of Nanog and Ssea1 were confirmed by immunocytochemistry and flow cytometry (Fig. 2d and Additional file 1: Figure S2B).

Next, we examined endogenous hepatic transcription network activation in iHepL cells. The qRT-PCR results showed that the endogenous expression of *Foxa1*, *Foxa2*, *Foxa3*, *Cebpa*, and *Hhex* were activated (Fig. 2e). *Hnf4a*, a core transcription factor involved in the hepatic cross-regulatory network and hepatocyte maturation [27], was endogenously expressed at high levels. iHepL cells significantly expressed genes such as *Afp*, *Slc4a2*, *MaoB*, *Fmo1*, or *Nat2*. In contrast, the expression of genes characteristic of mature hepatocytes was not homogeneous. While *Cyp7a1*, *Cyp2c39*, or *Ugt1a1* were expressed at significant levels compared to primary cultured mouse hepatocytes, other Cyp450 isozymes, *Tat*, *Slc10a1* (Ntcp), *Aat1*, or *Otc* were absent or hardly detectable. Hepatic-specific microRNA miR122, highly and specifically expressed during embryonic development of the liver [28], was expressed at hepatocyte-specific levels in iHepL cells.

**Fig. 3:**
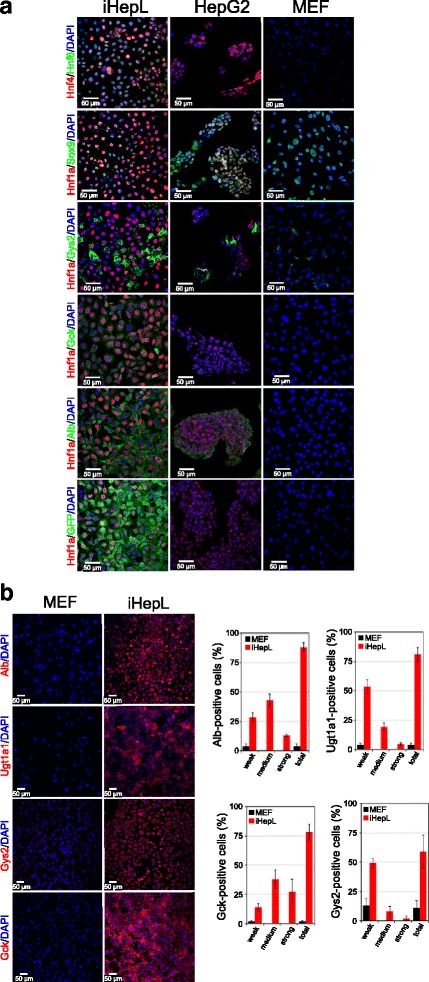
iHepL cells express hepatic genes. **a** Representative fluorescence images of HepG2, mouse embryonic fibroblasts (*MEF*), and hepatic progenitor-like (*iHepL*) cells immunostained with antibodies against multiple proteins. No differences were observed between the iHepL pool or clones 2, 6, and 15 (Additional file 1: Figure S3). Nuclei were stained with DAPI. **b** Representative fluorescence images at low magnification of MEF and iHepL cells immunostained for Albumin (Alb), Ugt1a1, Glucokinase (Gck), and Glycogen synthase 2 (Gys2). Nuclei were stained with DAPI. Bar diagrams in the *right panel* show the signal intensity distribution (strong, medium, or weak) in MEF or iHepL cells by the high content screening imaging station Scan® from Olympus. Data are represented as mean ± SD. Student’s *t* test was performed between iHepL and MEF. and statistical significance was *p* < 0.01 in all cases

Immunofluorescence analysis revealed high expression of multiple transcription factors (Hnf4, Hnf6, Hnf1, and Sox9) as well as hepatic enzymes such as Glucokinase (Gck), Glycogen synthase 2 (Gys2), and Albumin (Fig. 3a). The percentage of iHepL cells expressing those proteins as well as Ugt1a1 was between 60 and 90 %, showing a striking homogeneity of the cells (Fig. 3b). In fact, when individual iHepL cells were isolated and expanded, they displayed similar expression patterns (Additional file 1: Figure S3A). Interestingly, an average 99.4 ± 0.6 % of live iHepL cells were GFP-positive (Additional file 1: Figure S3B).

### iHepL cells perform basic hepatocyte-specific functions

**Fig. 4:**
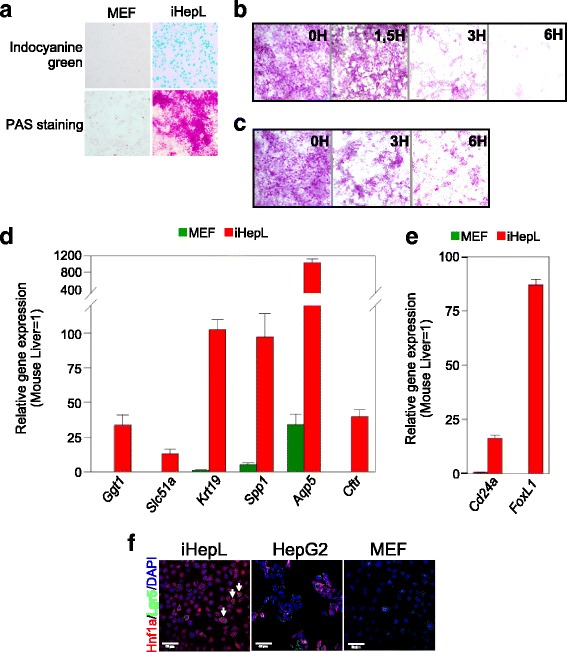
iHepL perform basal hepatocyte functions and express markers of bipotential hepatic progenitors. **a** Indocyanine green and periodic acid-Schiff (*PAS*) staining of hepatic progenitor-like (*iHepL*) cells and mouse embryonic fibroblasts (*MEF*). **b** PAS staining of iHepL treated with glucagon for 0, 1.5, 3, and 6 h. **c** PAS staining of iHepL cultured in glucose-free medium for 0, 3, and 6 h. **d, e** Total mRNA was extracted from MEF or iHepL cells and the expression of typical cholangiocyte (d) and hepatic-progenitor (e) markers was assessed by qRT-PCR and represented as relative gene expression normalized to total mouse liver. Data are represented as mean ± SD from iHepL cells (*n* = 6; three infections and two biological replicates each) and MEF (*n* = 3). Student’s *t* test was performed between iHepL and the other groups and statistical significance was *p* < 0.001 in all cases. **f** Representative fluorescence images of iHepL, HepG2, and MEF immunostained for Lgr5 and Hnf1a. No differences were observed between the iHepL pool or clones 2, 6, and 15 (Additional file 1: Figure S5). Nuclei were stained with DAPI

Having successfully proven the expression of the hepatic transcriptional program, we explored the ability of iHepL cells to perform specific hepatocyte tasks. Indocyanine green uptake, a clinically used test for basic liver function [29], was performed by most of the iHepL cells (Fig. 4a). PAS staining of the adherent cultures revealed that most of the cells stored glycogen and depleted it in response to glucagon (Fig. 4a and b). Moreover, when iHepL cells grown in complete media were changed to media without glucose, glycogen stores were also depleted (Fig. 4c). However, glycogen depletion was not followed by a significant secretion of glucose into the media, a role that depends on the activity of glucose-6-phosphatase. Similarly, iHepL cells could not generate urea when cells were incubated with ammonium chloride as a nitrogen source, an activity that relies on an active urea cycle (data not shown).

Hepatic Cyp450 are major enzymes accounting for drug detoxification expressed at high levels only in fully mature postnatal hepatocytes. iHepL cells expressed some phase I and phase II drug metabolic genes (*Cyp2c39*, *Ugt1a1*, *Fmo1*, and *Nat2*) at the mRNA level (Fig. 2a). To evaluate the acquisition of drug-metabolizing capabilities, we incubated iHepL cells with a cocktail of Cyp450 substrates [23] and monitored the formation of the metabolites by ultra-performance liquid chromatography-tandem mass spectrometry. iHepL cells were able to metabolize phenacetin, bufuralol, diclofenac, midazolam, mephenytoin, and bupropion, but the rate was between 30- and 500-fold lower than 24-h primary cultured mouse hepatocytes (Additional file 1: Figure S4). Remarkably, UDP-glucuronosyl transferase activity in iHepL cells (measured by glucuronidation of β-estradiol) was in the range of primary cultured hepatocytes.

The absence of mature hepatic functions prompted the investigation of whether iHepL cells were, in fact, hepatic progenitors or immature hepatocytes. Hepatic progenitors are bipotential, i.e., they can differentiate towards hepatocytes or cholangiocytes. Previous studies have shown that hepatic progenitors isolated from adult liver (Lgr5-positive [30], Foxl1-positive [15] and M + 133 + 26– cells [31]) or obtained by fibroblast reprogramming (iHepSC [9]) are enriched in duct-specific markers. Analysis by qRT-PCR confirmed that our iHepL cells express high mRNA levels of multiple duct cell markers (Fig. 4d). Moreover, iHepL cells also expressed *Cd24a* and *Foxl1*, specific lineage markers of bipotential hepatic progenitors in the adult liver (Fig. 4e). Lgr5 expression was not homogeneous, being only present in specific cells, even in cells expanded from a single cell-colony (Fig. 4f and Additional file 1: Figure S5). We concluded that iHepL cells do not functionally resemble mature hepatocytes, but bipotential hepatic progenitor cells.

### iHepL cells colonize the liver parenchyma and ducts of hepatectomized mouse after hepatic irradiation

In order to test the bipotentiality of iHepL cells in vivo, we performed intrasplenic transplantation into five mice subjected to liver irradiation and partial hepatectomy. Liver irradiation limits the proliferation of resident hepatocytes while hindering the immune response of the acceptor mice [32]. Partial hepatectomy provides the needed mitotic inducer in the donor hepatocytes. In order to track iHepL cells in vivo, we took advantage of maintained GFP expression from the bicistronic pMIGR1 retroviral vector (GFP-positive cells; Fig. 3a and Additional file 1: Figure S3).

**Fig. 5:**
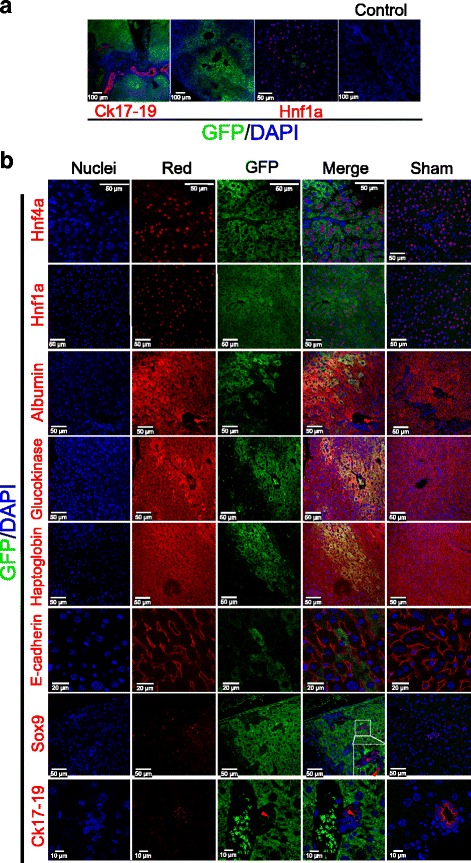
Repopulation of the liver of hepatectomized mouse after hepatic irradiation. Approximately 10^6^ iHepL cells (GFP-positive) were transplanted into partial hepatectomized and hepatic irradiated mice (*n* = 5). **a** Low magnification representative fluorescent images showing engraftment of iHepL cells in the mouse liver after 3 weeks. **b** Co-immunofluorescence staining of GFP with several hepatocyte and cholangiocyte markers. Nuclei were counterstained with DAPI (*blue*). Red blood cell auto-fluorescence is observed in some images. *GFP* green fluorescent protein

All five mice transplanted with iHepL survived and show different degrees of colonization, estimated to be between 5 % and 25 % based on GFP-positivity in serial sections (Additional file 1: Figure S6). Of note, our damage model provides no stimulus for expansion of the cells after engraftment, limiting the degree of colonization. Fluorescence above the general background was detected in big clusters, small clusters, and sometimes 1-3 isolated iHepL cells infiltrating into the surrounding mouse liver parenchyma (Fig. 5a). Contribution of iHepL cells to the bile duct network was limited and, commonly, big clusters of GFP-positive cells surrounding GFP-negative cells forming multiple duct structures could be observed (Fig. 5a). Immunostaining with Hnf4a, Hnf1a, Albumin, Haptoglobin, and Glucokinase antibodies confirmed the differentiation of iHepL cells toward the hepatocyte lineage (Fig. 5b). GFP-positive iHepL-derived cells fully integrated into the E-cadherin network of the liver parenchyma. Binucleation, a hallmark of hepatocyte maturation, was also commonly detected in GFP-positive cells. IHepL-derived cholangiocytes were identified by immunostaining with Sox9 and Ck17-19 antibodies (Fig. 5b). Approximately 2 % of iHepL-derived cholangiocytes were counted. Thus, iHepL cells display characteristics of bipotential hepatic cells and are able to differentiate into hepatocytes or cholangiocytes in vivo. Most of the iHepL cells in recipient mouse livers had stopped proliferation, similar to hepatocytes in wild-type mice [33], as assessed by the expression of the proliferation marker Ki67 and phospho-Histone H3 (Additional file 1: Figure S6 and data not shown).

**Fig. 6:**
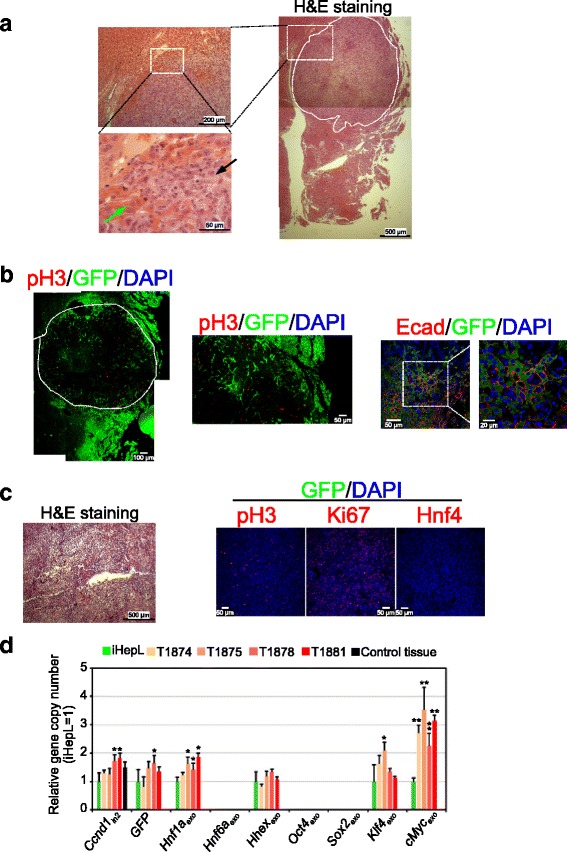
Tumorigenicity associated with iHepL cells. **a** Hematoxylin and eosin (*H&E*) staining of a liver section showing a cell mass after hepatic progenitor-like (*iHepL*) cell transplantation in the spleen. Tumor cells (*black arrow*) are less eosinophilic and the nuclei are abnormal in size and shape when compared to hepatocytes (*green arrow*). **b** Cells within the tumor that do not express green fluorescent protein (GFP) proliferated intensely and lost the characteristic E-cadherin staining. A *white dotted line* marking the borders of the cell mass is displayed. Co-labeling of GFP and phospho-Histone H3 (pH3) is not observed. **c** iHepL cells (1.5 × 10^6^/injection) in 150 μl DPBS were implanted subcutaneously in the right flank region of mice (*n* = 5). Upon sacrifice, primary tumors were removed, formalin-fixed, and histologically evaluated by H&E staining and immunostaining with antibodies against pH3, Ki67, and Hnf4. Nuclei were counterstained with DAPI (*blue*). **d** Relative gene copy number analysis. Genomic DNA isolated from iHepL cells in culture, embedded tumors, and embedded control liver mouse tissue was analyzed by qPCR using primers specific for intron 1 and intron 2 of *Ccnd1* gene, GFP, and fragments corresponding to lentiviral vectors pMIGR1-Hnf1a (*HNF1a*_exo_), pMIGR1-Hnf6a (*Hnf6a*_exo_), pMIGR1-Hhex (*Hhex*_exo_), pMSx-Oct4 (*Oct4*_exo_), pMXs-Sox2 (*Sox2*_exo_), pMXs-Klf4 (*Klf* *4*_exo_), and pMXs-cMyc (*cMyc*_exo_). Data were normalized to the level of Intron 1 of *Ccnd1* gene; a fragment of intron 2 from *Ccnd1* gene was used as a secondary normalization control. Then values for each sample were normalized to the value for iHepL cells. *p < 0.05; **p < 0.005 (compared to iHepL cells)

In hematoxylin-eosin stained sections, no major abnormalities were observed in most of the mice, except for a dysplastic cell mass found in one of the livers. Nuclei within the cell mass were very heterogeneous in size and shape, and cells infiltrated to the liver parenchyma (eosinophilic, orange staining), suggesting a cell malignancy. The presence of dysplasia in bile cholangioles suggest that the origin of the tumor might be the bile duct tree (Fig. 6a). It is noteworthy that the cells within the tumor were GFP-negative (Fig. 6b). Tumor cells were highly proliferative based on phospho-Histone H3 (pH3) immunostaining; however, GFP-positive cells present in the surrounding tissue were not proliferating. Tumor cells were also negative for E-cadherin. In fact, an E-cadherin staining pattern was only maintained in GFP-positive cells found at the interface between normal and tumorigenic tissue. In conclusion, the absence of mature ectodermal, mesodermal, and endodermal structures, the lack of encapsulation, and the high proliferation rate of the cells exclude that tumor cells were a teratoma.

The absence of GFP in the tumor cells did not clarify the cellular origin of the tumor. In order to investigate whether the tumor originated from our iHepL cells, we ran conventional tumorigenic analysis by subcutaneous injection in four mice. After 45 days, all the animals developed a solid dysplastic cell aggregate mainly composed of highly proliferative undifferentiated cells (Fig. 6c). Again, cells were GFP-negative, recapitulating the features of the cell mass found in one of the livers. We could not detect any evidence of mature ectodermal, mesodermal, or endodermal structures (most frequently skin, hair follicles, sebaceous and sweat glands, cartilage, adipose tissue, and glia) characteristic of teratomas typically originated from embryonic stem cell implanted in nude mice. To get a deeper insight into the tumor cell origin, we performed a comparison of transgene copy number in genomic DNA isolated from tumors and iHepL (Fig. 6d). Fifty percent or more of the tumors contain the same copy number for GFP, *Hnf6a*_exo_, *Hhex*_exo_, *Oct4*_exo_, *Sox2*_exo_, and *Klf4*_exo_ than iHepL cells (Additional file 1: Table S6). Moreover, iHepL and tumor cells do not contain the transgenes for *Oct4* and *Sox2* in their genomic DNA, confirming the lack of exogenous expression. These analyses exclude the possibility that partially pluripotent iPSC are the origin of tumors and a possible transition through a transient pluripotent state before committing to the hepatic lineage.

## Discussion

Cell reprogramming technology holds great promise as a potential strategy in cell replacement therapies. However, the possible tumorigenic potential of reprogrammed cells has also raised concerns about their clinical use. Several authors have reported the generation of iHep cells without tumorigenic features [5, 7, 9]. Here, we report the direct lineage conversion into hepatic progenitor-like cells (iHepL) with the use of pluripotency factors which show tumorigenicity when transplanted in vivo. Tumor cells and iHepL cells do not contain transgenes for *Oct4* or *Sox2*, pointing at *Klf4* and *Myc* as probably responsible for induced tumorigenicity and excluding rare partially pluripotent cells as responsible for tumor formation. Our work highlights that the risk of tumorigenesis in direct lineage conversion using pluripotency factors should be examined with the same rigor as in the case of iPSC-derived lineage cells. On the other hand, recent reports have indicated that the inclusion of pluripotent transcription factors during direct somatic lineage conversion cause cells to go through a transient pluripotent state during cardiac and neural somatic cell transdifferentiation [34, 35]. The absence of *Oct4* and *Sox2* transgenes in iHepL genomic DNA suggests that MEF directly committed into the hepatocyte lineage, since Oct4 and Sox2 are essential factors for pluripotent reprogramming [18]. Moreover, a recent report has found that *Klf4* and *Myc* dramatically accelerate direct hepatic reprogramming, probably by promotion of a mesenchymal-to-epithelial transition [36].

iHepL cells are able to perform basic hepatic functions such as glycogen storage or indocyanine green uptake, but fail to perform functions characteristic of mature hepatocytes such as urea cycle or high Cyp450-dependent drug metabolism. Knowing this, it is striking that the mRNA level of *Cyp2c39* in iHepL cells is 43 % of that of 24-h primary hepatocytes in culture, while the actual activity is 75-fold lower (0.01 %). Primary hepatocytes in culture lose the transcription of genes characteristic of mature hepatocytes within the first 2–4 h [37]. In fact, when we measured the expression of *Cyp2c39* in mouse hepatocytes cultured for 4, 24, 48, and 72 h the levels dropped 97 % in the initial 24 h compared to mouse liver (Additional file 1: Figure S7A). However, Cyp2c39 activities remained fairly stable during the first 24–48 h (Additional file 1: Figure S7B). This is probably due to the longer protein half-life compared to its mRNA [38].

Recently, the direct conversion of fibroblasts into hepatic-progenitor cells (iHepSC) able to differentiate in vivo into hepatocytes and cholangiocytes was reported [9]. iHepSC and hepatic progenitor cells isolated from adult livers (Lgr5-positive cells [30] and Foxl1-positive cells [15]) shared the expression of multiple markers, including genes specifically expressed in duct/cholangiocytes. iHepL cells display high levels of duct cell markers together with immature hepatocyte markers. The progenitor nature of our iHepL cells is endorsed by the elevated expression of *Foxl1* and *Cd24a* mRNA (20-fold and 85-fold compared to whole liver) and Lgr5 protein, which are markers for bipotential progenitor cells isolated from adult liver [14, 15].

Progenitor features of iHepL cells were confirmed in transplantation studies. Engrafted iHepL cells were identified by GFP expression, since directly converted iHep cells do not completely silence the retroviral gene [5, 7]. iHepL cells differentiated into hepatocytes and cholangiocytes in vivo. The presence of iHepL-derived hepatocytes (parenchyma) and cholangiocytes (bile ducts) differs significantly. While we estimated an engraftment of 5–25 % in the liver parenchyma (GFP-positive hepatocytes vs total), the engraftment in bile ducts was around 2 %. This result suggests that iHepL cell bipotentiality is biased towards the hepatocyte lineage, or that our in vivo model favors regeneration of the duct network with resident cholangiocytes/progenitors. Previous reports of iHep cells derived from MEF have never found any evidence of cell fusion when transplanted into mouse [5, 7, 9]. Nevertheless, Y chromosome staining in male livers transplanted with female iHepL cells would be necessary to exclude the possibility of cell fusion.

Cell transplantation studies using the fumarylacetoacetate hydrolase-deficient mice (Fah–/–) favors the proliferation/survival of engrafted hepatocyte into the liver parenchyma but not cholangiocytes [9]. We have used two-thirds hepatectomy and irradiation as an in vivo model since this provides an optimal niche for parenchymal (hepatocyte) and non-parenchymal (cholangiocyte) cell engraftment [39, 40]. Moreover, the engraftment of the exogenous cells in the irradiated liver lobe constitutes by itself a rescued experiment because this lobe would atrophy if no exogenous cells are supplied, while there is no proliferation/survival advantage to engrafted cells, limiting the extension of liver colonization.

One out of five transplanted mice developed a GFP-negative cell mass juxtaposed to the liver. In addition, when cells were subcutaneously injected in nude mice, they generated dysplastic cell masses composed of GFP-negative, undifferentiated, highly proliferative cells. Although a combination of qRT-PCR, immunocytochemistry, and flow cytometer analysis did not detect the expression of *Oct4*, *Sox2*, *Nanog*, or *Ssea1*, it is still feasible that these cell masses could be teratomas originating from rare GFP-negative partially pluripotent cells contaminating iHepL cells. However, the solid nature of the cell mass (not encapsulated, not cyst-like), the absence of any mature structure (endodermal, mesodermal, or ectodermal), and the high proliferation rate of the cells lets us conclude that it is not a teratoma but a tumor malignancy. Still, tumors could have been originated by rare tumorigenic GFP-negative cells contaminating our iHepL cells. Again, we favor an alternative hypothesis, since 50 % or more of the tumors contain the same copy number for GFP, *Hnf6a*_exo_, *Hhex*_exo_, *Oct4*_exo_, *Sox2*_exo_, and *Klf4*_exo_ as iHepL cells. All tumors contain three-fold the number of copies of the *Myc* transgene. If rare tumorigenic GFP-negative *Myc*^high^ cells were contaminating our iHepL cultures, GFP-positive cells would dilute out after multiple cell passes, something we never observed (Additional file 1: Figure S3B depicts the analysis of p20 iHepL).

Based on the fact that tumor cells were GFP-negative, but contain the same number of copies of the GFP transgene, we hypothesize that cell masses are not teratomas originating from partially pluripotent cells, but that malignant tumors originated from GFP-positive iHepL cells that lost expression of hepatic factors (co-expressed with GFP from a bicistronic cassette) triggering the tumorigenicity of *Myc* [41].

## Conclusions

Our study highlights the dangers of using pluripotency factors, in particular *Klf4* and *Myc*, in cell reprogramming due to their intrinsic oncogenic properties. The expression of hepatic cell fate mediators triggered reprogramming into cells with gene expression profiles similar to that of other hepatic-progenitor cells isolated from adult liver, able to differentiate in vivo into hepatocytes and cholangiocytes. However, we obtained evidence that silencing of hepatic cell fate mediators such as *Hhex* and/or *Hnf1a* result in tumorigenicity. Thus, extensive tumorigenic studies are essential in any reprogramming endeavor even if no tumors are detected in the in vivo engraftment experiments.

## Abbreviations

7-AAD, 7-amino actinomycin D; DMEM, Dulbecco’s modified Eagle’s medium; EGF, epidermal growth factor; FBS, fetal bovine serum; FGF, fibroblast growth factor; GFP, green fluorescent protein; HCM, hepatocyte conditioned medium; HGF, hepatocyte growth factor; iHepL, hepatic progenitor-like; iHepSC, induced hepatic stem cells; iPSC, induced pluripotent stem cells; LC-MS/MS, liquid chromatography mass spectrometry; LIF, leukemia inhibitory factor; MEF, mouse embryonic fibroblasts; OSK/M, Oct4, Sox2, Klf4/Myc; PAS, periodic acid-Schiff; PBS, phosphate-buffered saline; PCR, polymerase chain reaction; PH, partial hepatectomy; qPCR, quantitative polymerase chain reaction; TGF, transforming growth factor; VEGF, vascular endothelial growth factor

## Additional file

Additional file 1: Supplementary information. (PDF 923 kb)
