# Supplementary material for: Silencing of hepatic fate-conversion factors induce tumorigenesis in reprogrammed hepatic progenitor-like cells
Source: Stem Cell Res Ther. 2016 Jul 27;7:96. doi: 10.1186/s13287-016-0349-5 (PMC4962402; doi:10.1186/s13287-016-0349-5)
Supplement: Additional file 1: — Supplementary information. (PDF 923 kb) [file 13287_2016_349_MOESM1_ESM.pdf]

A

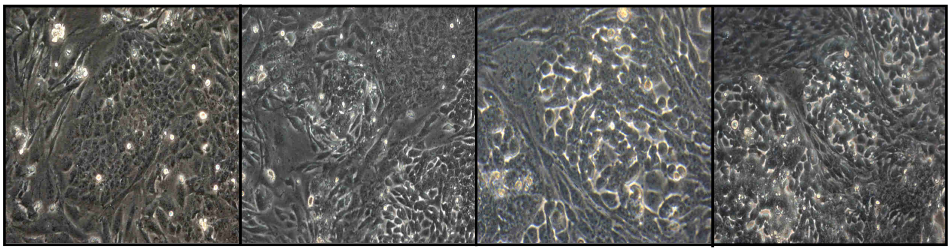

B

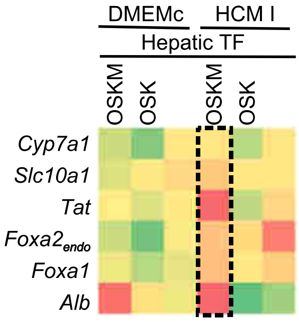

SUPPLEMENTARY FIGURE S1
(Serrano et al)

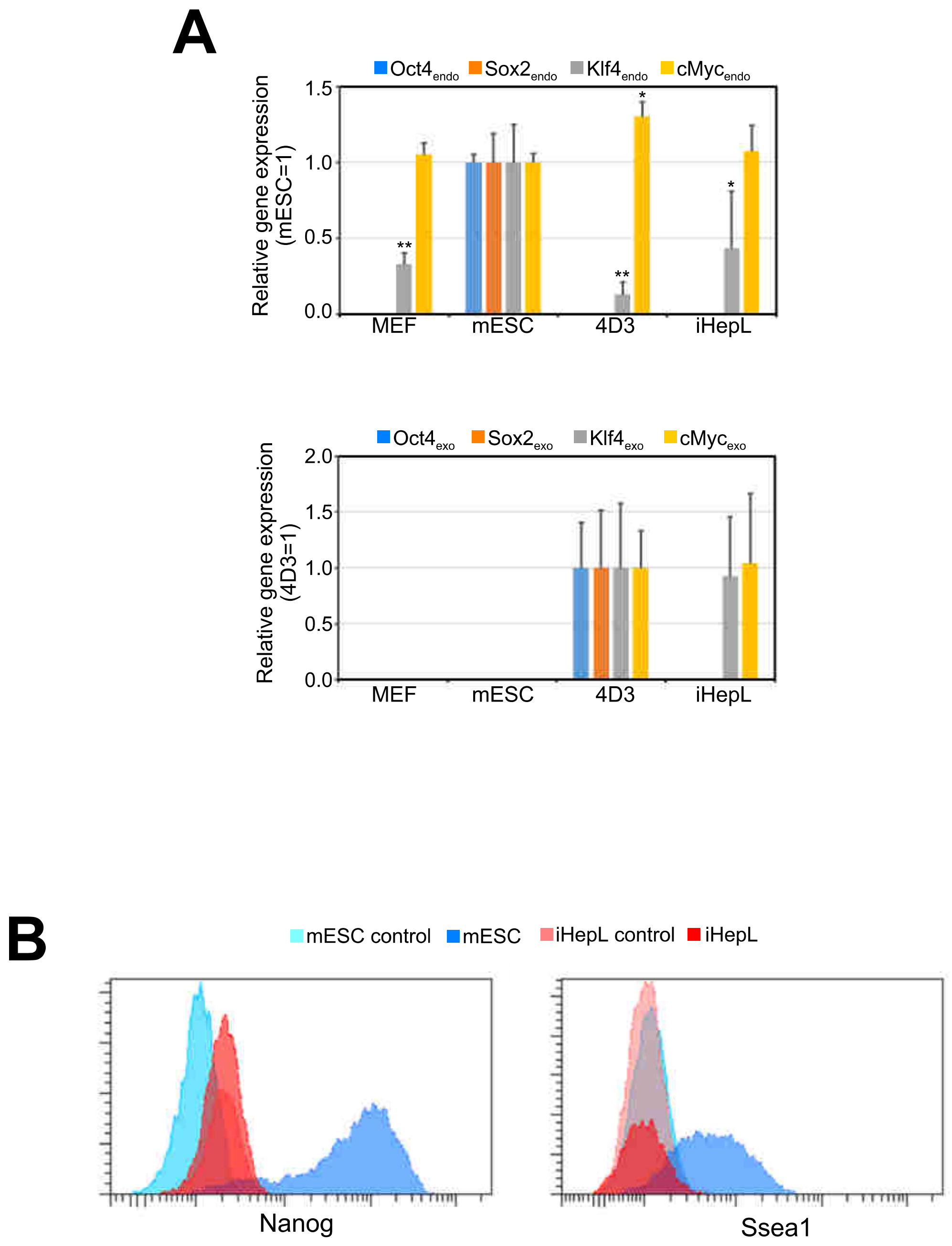

SUPPLEMENTARY FIGURE S2
(Serrano et al)

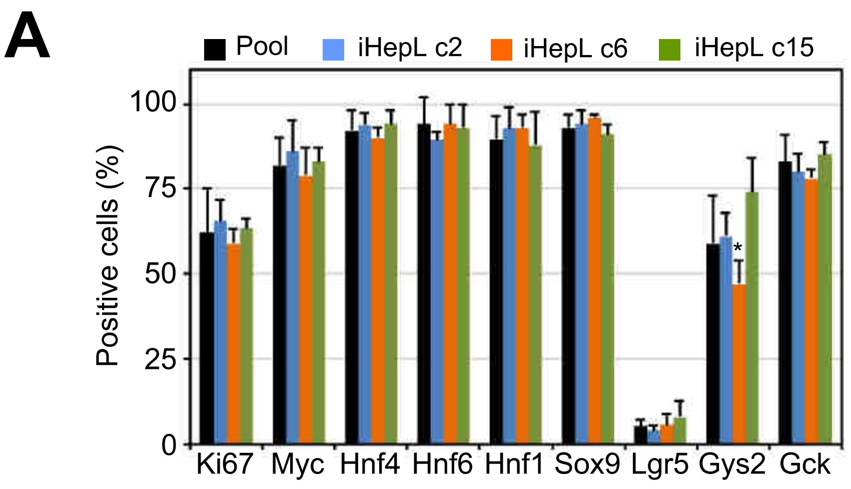

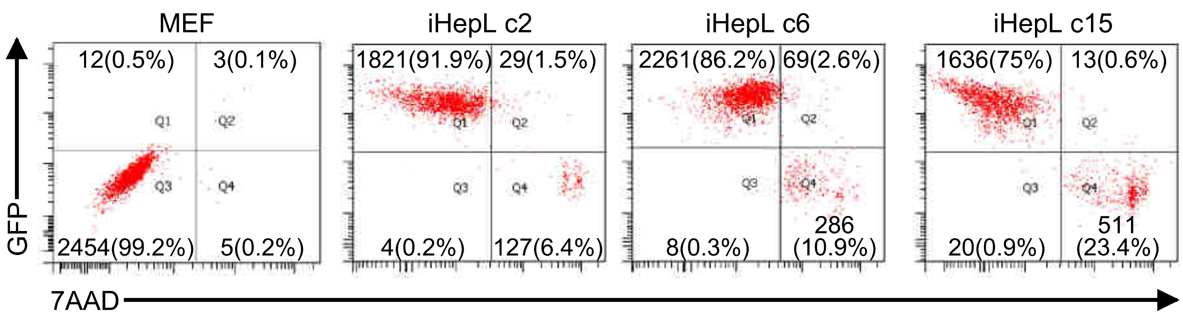

SUPPLEMENTARY FIGURE S3
(Serrano et al)

A

B

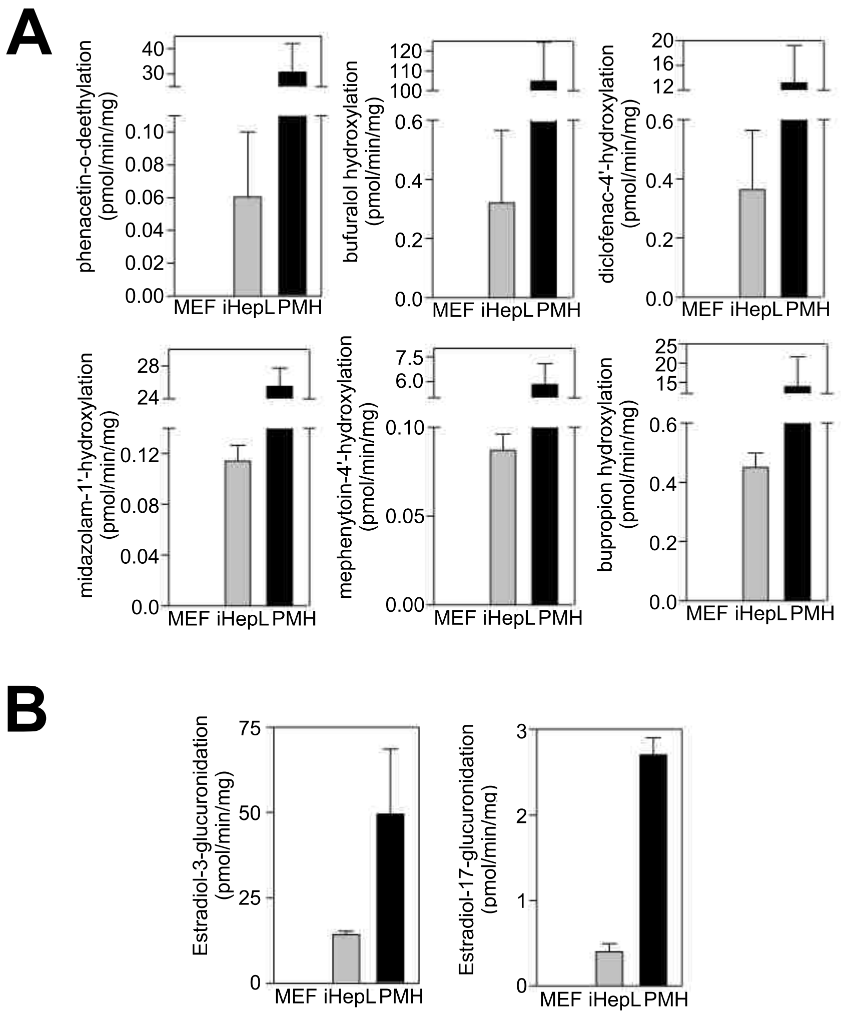

SUPPLEMENTARY FIGURE S4
(Serrano et al)

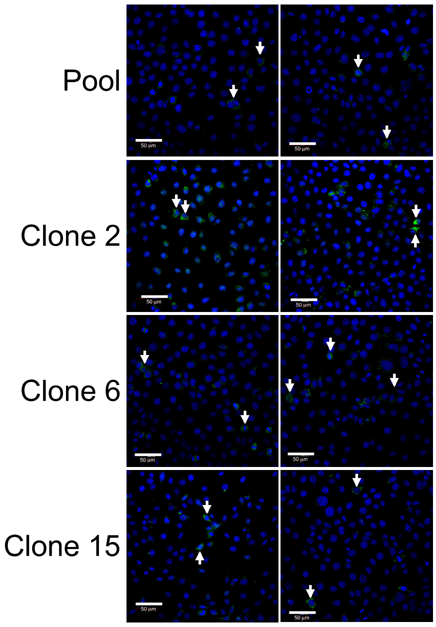

SUPPLEMENTARY FIGURE S5
(Serrano et al)

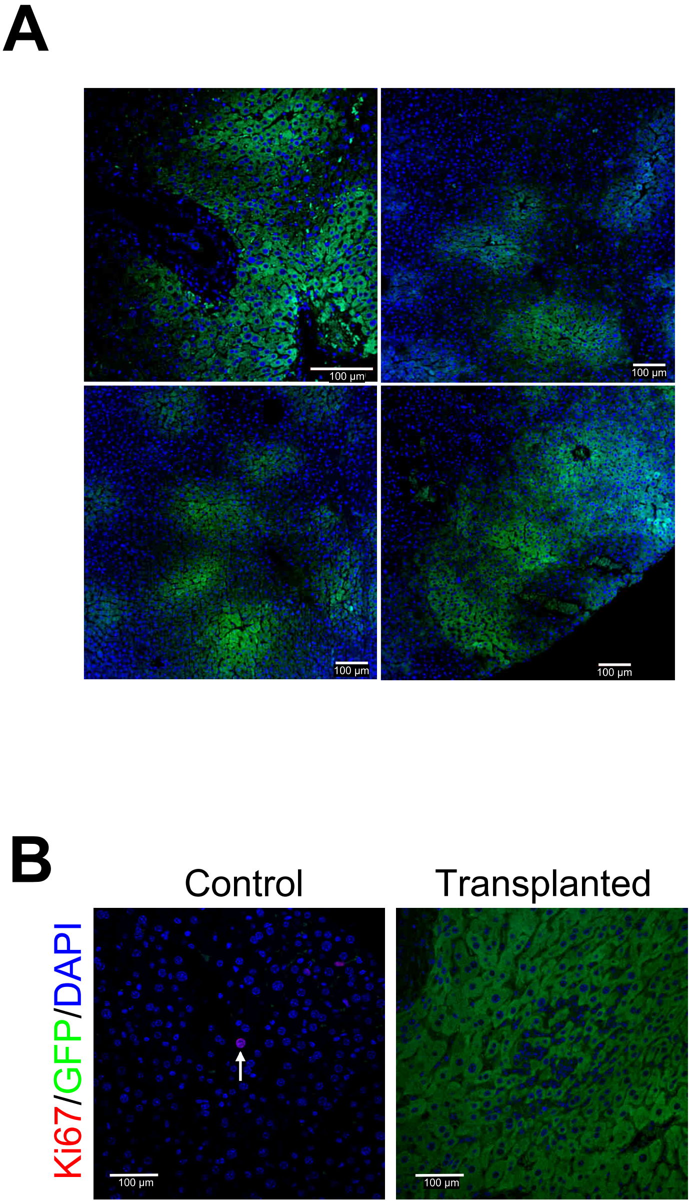

SUPPLEMENTARY FIGURE S6
(Serrano et al)

A

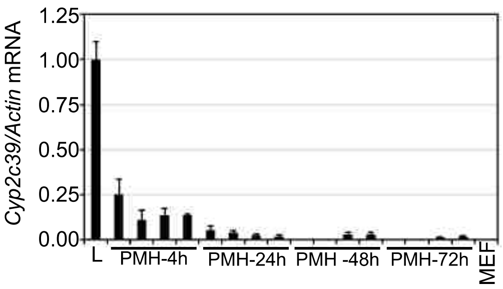

B

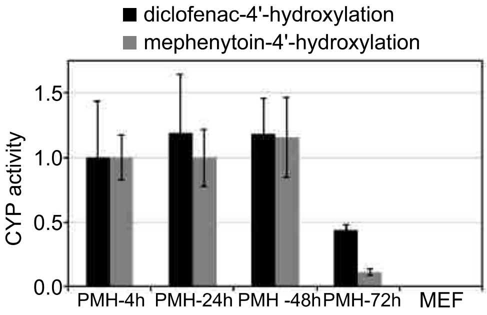

SUPPLEMENTARY FIGURE S7
(Serrano et al)

Supplementary Figure S1. Formation of epithelial colonies in infected MEF. A. Phase contrast photographs of several epithelial colonies (7 days after infection) formed in MEF infected with the retroviral mixture. **B.** Heat map depicting relative expression levels of the mRNAs analyzed in Figure 1B. Red: higher expression; green: lower expression.

Supplementary Figure S2. iHepL cells do not express endogenous pluripotency factors Oct4 and Sox2. A. Expression levels of endogenous and exogenous (transgenic) transcripts for Oct4, Klf4, Sox2 and c-Myc were measured by qRT-PCR in total RNA extracted from MEF, mESC (CGR8 strain), iHepL and 4D3 (MEF infected with lentiviral vectors expressing OSKM for 3 days) cells. Data is represented as mean ± s.d. from MEF (n=3), mESC (n=3), iHepL cells (n=6) and 4D3 (n=3). Student´s t test was performed between mESC (upper panel) or 4D3 (lower panel) and the other groups; * $p<0.05$; ** $p<0.01$. **B.** Expression of Nanog and Ssea1 in mESC (CGR8 strain) and iHepL analyzed by flow cytometry. Controls were incubated with normal serum instead of the primary antibody.

Supplementary Figure S3. Characterization of iHepL isolated clones. A. Original unselected pool of iHepL cells and isolated clones from three different infections (see below) were immunostained as shown in Figure 3. The percentage of positive cells was calculated using the CellC software {Selinummi, 2005 #551} and high content screening imaging station ScanˆR from Olympus. Total cell number ranged from 1000 to 4000. Total cell numbers were estimated with DAPI staining. No statistically significant difference was found between the pool and any of the clones except for Gys2 immunostaining in clones 6 and 15, $p<0.05$. **B.** Cells (MEF and iHepL clones) were incubated for 30 min in media containing 50 μg/ml of 7-amino actinomycin D (7-AAD) and analyzed for viability (7AAD-negative) and GFP expression by FACSCanto Flow Cytometer (Becton Dickinson). FACSCanto. iHepL c2: clone 2 from infection #1, iHepL c6: clone 6 from infection #2 and iHepL c15: clone 15 from infection #3.

Supplementary Figure S4. Limited Cyp450 activities in iHepL cells. A. Cells were incubated with a mixture of 8 substrates and the corresponding metabolites measured by LC-MS. Formation of 6-hydroxychlorzoxazone and 7-hydroxycoumarin were not detected. **B.** Formation of estradiol-3-glucuronide (Ugt1a1) and estradiol-17-glucuronide (Ugt2b) in iHepL incubated with estradiol. Data is represented as mean ± s.d. from iHepL cells (n=6) and PMH (n=3). Student´s t test was performed and statistical significance was in all cases $p<0.001$.

Supplementary Figure S5. Expression of hepatic progenitor marker Lgr5 in pool iHepL and isolated clones. Representative fluorescence images of iHepL cells immunostained with an antibody against Lgr5. Two images per cell type are shown. Nuclei were made visible with DAPI.

Supplementary Figure S6. iHepL cells (GFP-positive) engraftment in mouse livers. A. Low magnification representative fluorescent images showing engraftment of iHepL cells in the mouse liver after three weeks. **B.** Serial sections of transplanted livers were stained by Ki67. Only sporadic positive cells (arrow in control) were detected among resident and transplanted cells. Nuclei were made visible with DAPI.

Supplementary Figure S7. mRNA and activity decay in cultured hepatocytes. A. Total RNA was extracted from cultured primary mouse hepatocytes (PMH) at the indicated times and the expression of *Cyp2c39* was assessed by qPCR and represented as relative gene expression normalized to total mouse liver (L). Data are represented as mean ± s.d. (L, n=5; MEF, n=3; PMH, n=4). **B.** Graph showing Cyp450 activity in PMH. Diclofenac and mephenytoin 4 hydroxylation by primary cultured mouse hepatocytes (n=4) at different culture times. Cells were incubated with a diclofenac and mephenytoin and the corresponding metabolites measured by LC-MS.

Supplementary Table S1. Primers used for cloning

Primer	Sequence 5'-3'
pMIGR1-Hhex-XhoI-F	AACTCGAGATGCAGTTCCCGCACCCGGGG
pMIGR1-Hhex-EcoRI-R	GGGAATTCTCATCCAGCATTAAAGTAGCC
pMIGR1-Hnf1a-XhoI-F	AACTCGAGACCATGGTTTCTAAACTGAGCCAG
pMIGR1-Hnf1a-EcoRI-R	GGGAATTCTTACTGGGAGGAAGAGGCCAT
pMIGR1-Hnf6a-XhoI-F	AACTCGAGACCATGAACGCGCAGCTGACCATG
pMIGR1-Hnf6a-EcoRI-R	GGGAATTCTCATGCTTTGGTACAAGTGC

Supplementary **Table S2. Retroviral plasmids used in this study.**

Plasmid	Backbone	Developed by	Addgene
pMSx-Oct4	pMXs	Dr Shinya Yamanaka	#13366
pMXs-Sox2	pMXs	Dr Shinya Yamanaka	#13367
pMXs-Klf4	pMXs	Dr Shinya Yamanaka	#13370
pMXs-cMyc	pMXs	Dr Shinya Yamanaka	#13375
pBabe-Foxa2	pBabe-PURO	Dr Ken Zaret	
pBabe-Gata4	pBabe-PURO	Dr Ken Zaret	
pBabe-Foxa2	pBabe-PURO	Dr Ken Zaret	
pMIGR1-Hhex	pMIGR1-IRESGFP	In house	
pMIGR1-Hnf1a	pMIGR1-IRESGFP	In house	
pMIGR1-Hnf6a	pMIGR1-IRESGFP	In house	

Supplementary **Table S3. Primers used for qRT-PCR**

Transcript	Forward (5'-3')	Reverse (5'-3')	Size (bp)
Albumin	gcatgggcagtagctcgcct	ggcaaggtccgccctgtcat	150
Slc10a1	tggctacctcctccctgatgcc	cctgtttccatgctgatggtgcgt	108
Cyp7a1	caacgggttgattccatacc	atttccccatcagtttgcag	118
Foxa1	acgaggagccccatcgagcc	accaggttggccatgcagaca	124
$Foxa2_{endo}$	cgaacaaagcgggcctggat	cctctcatttcccttgtccccagt	142
Foxa3	ctctattcccgctctctgctt	ggcagttaccacagtagccaa	179
Cebp	ccatgccgggagaactctaac	tggaggtgactgctcatcgg	86
$Hhex_{endo}$	cccagcctctcaggaagacccc	gtccggtctctaaacatggccga	125
$Hnf4a_{endo}$	tgaatcctacaagctcctgcc	taggttactcccaggtgctct	164
$Oct4_{endo}$	gtggacctcaggttggactgg	ttctgcagggctttcatgtcc	199
$Sox2_{endo}$	tagagctagactccgggcgatga	ttgctttaaacaagaccacgaaa	296
$Klf4_{endo}$	ccaacttgaacatgcccggactt	tctgcttaaaggcatacttggga	498
$cMyc_{endo}$	tgacctaactcgaggaggagctggaatc	aagtttgaggcagttaaaattatggctgaagc	142
$Oct4_{exo}$	ttgggctagagaaggatgtggttc	ttatcgtcgaccactgtgctg	421
$Sox2_{exo}$	ggttacctcttcctcccactccag	ttatcgtcgaccactgtgctg	376
$Klf4_{exo}$	gcgaactcacacaggcgagaaacc	ttatcgtcgaccactgtgctg	406
$cMyc_{exo}$	cagaggaggaacgagctgaagcgc	ttatcgtcgaccactgtgctg	426
Afp	gttgccaaggaaactcgctg	ggtttgacgccattctctgc	163

MaoB	acctagcaagcagcatgagc	gtaagtcctgcctcctacacg	146
Fmo1	ttaccaccgccaagtgtcat	gatgctcagagccagtcgtg	195
Nat2	cccggttgcagtcctggtag	tagcccgtatctggtgctga	132
Cyp1a2	cccctgcccttcagtggtaca	agcacgtccccatactgctg	246
Cyp2e1	gacgtgcggaggttttccct	ttgcagggtgcacagccaat	164
Cyp2c39	ggccagtgatgcctgatcacaat	aatcgggaaaggagtggggc	159
Cyp3a11	acctgggtgctcctagcaat	acagcaaggagaggcgtttgac	221
Ugt1a1	tgggaggctgttagtgttccc	accacgcgcagcagaaaaga	249
Tat	accttcaatcccatccga	tcccgactggataggtag	205
Aat1	atatccccttggctcccat	ttcagcttgctgtcccagag	96
Otc	gctgcgtctgactggacatt	gcttctggagcacaggtgag	175
Cdh1	aacaactgcatgaaggcgggaatc	cctgtgcagctggctcaaatcaaa	246
Ocln	cctccaatggcaaagtgaatggca	tgtttcatagtggtcagggtccgt	227
Snai1	ttgtgtctgcacgacctgtggaaa	tcttcacatccgagtgggtttgga	167
Zeb1	tgctcacctgcccgtattgtgata	agtgcacttgaacttgcggtttcc	224
Thy1	acccgagcacacgtaccgct	tgccgccacacttgaccagc	190
Nanog	ccactagggaaagccatgcgc	aggaacctggctttgccctgac	135
Lin28	ggagtgagcggcggccaaaa	gctggggtggcagcttgcat	116
Ggt1	ttgcctatgccaagaggacc	ctgggtgggtggtttcatca	132
Slc51a	tggccatccttttccgtcaa	agcgaacaagcctcataccc	286
Krt19	gtgccaccattgacaactcc	aatccacctccacactgacc	287
Spp1	agccacaagtttcacagcca	aggaactgtgtttttgcctct	147
Aqp5	gtcacactgggccatcttgt	cgatcggtcctacccagaag	130
CD24a	ttcgcatggtcacacactga	acacacacagtagcttcggg	124
Cftr	ttggcccgatcagttctcag	gcctgaagggagtcgtactg	221
Actin	ccaccatgtacccaggcatt	agggtgtaaaacgcagctca	250

Supplementary Table S4. Primers used for genomic qPCR

Transcript	In plasmid	Forward (5'-3')	Reverse (5'-3')	Size (bp)
$Ccnd1_{In1}$		tggatttggaggaacctgtc	ccctgagtctcctgctgaac	85
$Ccnd1_{In2}$		atcctgtaggccaggtgatg	aggctcaagggcttttaagg	81
GFP	pMIGR1-IRES-GFP derived	tatatcatggccgacaagca	gttgtggcggatcttgaagt	60
$Hnf1_{exo}$	pMIGR1-Hnf1a-IRES-GFP	atggcctcttcctcccagta	acaccggccttattccaag	80
$Hnf6a_{exo}$	pMIGR1-Hnf6a-IRES-GFP	tcaagcacttgtaccaaagca	accggccttattccaagc	81
$Hhex_{exo}$	pMIGR1-Hhex-IRES-GFP	tcgagggggataaaggctac	acaccggccttattccaag	94
$Oct4_{exo}$	pMXs-Oct4	gtggtggtacgggaaatcac	cgaagtctgaagccaggtgt	82
$Sox2_{exo}$	pMXs-Sox2	gtggtggtacgggaaatcac	ttcagctccgtctccatca	71
$Klf4_{exo}$	pMXs-Klf4	gtggtggtacgggaaatcac	gctggacgcagtgtcttct	146
$cMyc_{exo}$	pMXs-cMyc	gtggtggtacgggaaatcac	agggctgtacggagtcgtag	127

Supplementary Table S5. Antibodies used in this study.

Antibody	Raised in	Dilution	Reference	Use
Albumin	Mouse	1/100	AB1455; Merck Millipore	IC, IHC
Ck17-19	Rabbit	1/50	12434; Cell Signalling Technology	IC, IHC
E-cadherin	Rabbit	1/500	3195; Cell Signalling Technology	IC, IHC
GFP	Goat	1/200	Ab6673; Abcam	IC, IHC
Glucokinase	Rabbit	1/50	Orb38697; Biorbyt	IC, IHC
Haptoglobin	Rabbit	1/50	Orb28600; Biorbyt	IHC
Gys2	Rabbit	1/250	HPA039482; Sigma	IC
Hnf1	Rabbit	1/200	8986; Santa Cruz Biotechnology	IC, IHC
Hnf1	Goat	1/200	6547; Santa Cruz Biotechnology	IC, IHC
Hnf4	Goat	1/200	6556; Santa Cruz Biotechnology	IC, IHC
Hnf6	Rabbit	1/200	13050; Santa Cruz Biotechnology	IC
Ki67	Rabbit	1/200	9106; Thermo ScientICic	IC, IHC
Lgr5	Rabbit	1/50	Ab75732; Abcam	IC
Myc	Rabbit	1/200	Ab32072; Abcam	IC, IHC
Nanog	Rabbit	1/100	REC-RCAB0001P; Cosmo Bio Ltd	IC, FC
Oct4	Rabbit	1/400	2840; Cell Signalling Technology	IC, FC
pH3	Rabbit	1/200	9701S; Cell Signalling Technology	IC, IHC
Sox9	Rabbit	1/100	AB5535; Merck Millipore	IC, IHC
Ssea1	Mouse	1/100	4744; Cell Signalling Technology	IC, FC
Ugt1a1	Rabbit	1/750	AV44294; Sigma	IC

Supplementary Table S6. Statistical significance of relative transgene copy number. The following table shows the statistical significance calculated by unpaired Student's t-test between iHepL cells and each of the samples included in Figure 6D. A fragment of intron 2 from Ccnd1 gene was used as a secondary normalization control.

	**TARGET GENES**							
	**Ccnd1in2**	**GFP**	**Hnf1a$_{exo}$**	**Hhex$_{exo}$**	**Oct4$_{exo}$**	**Sox2$_{exo}$**	**Klf4$_{exo}$**	**Myc$_{exo}$**
**T1874**	0.149	0.528	0.059	0.445	N.D.	N.D.	0.102	<u>0.000</u>
**T1875**	0.188	0.102	<u>0.001</u>	0.386	N.D.	N.D.	<u>0.019</u>	<u>0.001</u>
**T1878**	<u>0.010</u>	<u>0.038</u>	<u>0.006</u>	0.144	N.D.	N.D.	0.297	<u>0.001</u>
**T1881**	<u>0.005</u>	0.203	<u>0.000</u>	0.778	N.D.	N.D.	0.687	<u>0.000</u>
**Control**	0.065	N.D.	N.D.	N.D.	N.D.	N.D.	N.D.	N.D.

N.D: not detected
